# Clinical results following colonic resection for ulcerative colitis in elderly individuals (elderly-onset vs. nonelderly onset)

**DOI:** 10.1186/s12893-022-01664-2

**Published:** 2022-06-03

**Authors:** Ryuichi Kuwahara, Hiroki Ikeuchi, Toshihiro Bando, Yoshiko Goto, Yuki Horio, Tomohiro Minagawa, Motoi Uchino

**Affiliations:** 1 Department of Gastroenterological Surgery, Hyogo College of Medicine, Nishinomiya, Japan; 2 Department of Gastroenterological Surgery, Hyogo College of Medicine, 1-1, Mukogawa-cho, Nishinomiya, Hyogo 663-8501 Japan

**Keywords:** Ulcerative colitis, Elderly, Nonelderly, Operation

## Abstract

**Background:**

The incidence of ulcerative colitis (UC) is increasing, but there are few reports comparing elderly UC patients undergoing colectomy for elderly-onset UC (EO) and nonelderly-onset UC (NEO). The aim of this study was to analyze the differences between EO and NEO patients who underwent UC-related surgery.

**Methods:**

We identified 1973 patients with UC who underwent colectomy at Hyogo College of Medicine between January 1, 1984, and December 31, 2018. Only patients aged 65 years old and older who underwent colectomy were enrolled in this study (n = 221, 11.2%), and their clinical records were retrospectively reviewed. Patients were divided into two groups according to their age at disease onset: those with onset at younger than 60 years old (NEO) and at 60 years old or older (EO).

**Results:**

In the 221 UC patients who underwent colectomy at 65 years old or older, there were 155 cases of EO and 66 cases of NEO. The main surgical indication in NEO patients was colitis-associated cancer/dysplasia (32/66, 47%). In contrast, refractory to medical treatment was the leading cause of surgery in EO patients (80/155, 52%). The distributions of surgical indications were different between the two groups (p < 0.01). The preoperative daily dose of steroids was significantly higher in the EO group than in the NEOgroup (0 mg vs. 10 mg, p < 0.01). The rates of immunosuppressant, infliximab (IFX) and adalimumab use did not differ significantly between the groups. Significantly more patients underwent emergency surgery in the EO group than in the NEO group (14% vs. 35%, p < 0.01). The proportions of patients with postoperative morbidity (Clavien-Dindo grade III or higher) were 17.4% (27/155) in the EO group and 13.6% (9/66) in the NEO group. There was no significant difference between the two groups (p = 0.48). The prognosis of the EO patients who underwent UC-related emergency surgery was worse than that of the NEO patients (p < 0.01). In the EO group, 8 (14.8%) of 54 patients died within 30 postoperative days, while there were no deaths in the NEO group.

**Conclusion:**

Among elderly UC patients undergoing UC-related surgery, EO patients undergoing emergency surgery had very poor outcomes, and the mortality rate was 14.8%. In such cases, it is important for physicians and surgeons to begin communication at an early stage so that the optimal surgical timeframe is not missed.

**Supplementary Information:**

The online version contains supplementary material available at 10.1186/s12893-022-01664-2.

## Background

Ulcerative colitis (UC) is a refractory disease with an unknown cause. While considered a condition primarily affecting young adults, UC can develop at any age, including old age. In fact, UC has bimodal incidence peaks, with the second peak occurring between the ages of 50–80 years [1–6].

Elderly patients with UC can be divided into two groups: those with elderly-onset UC (EO) and those with nonelderly-onset UC (NEO). The largest population-based study evaluating the natural history of EO was performed in France and reported a milder disease course in elderly-onset inflammatory bowel disease (IBD) patients compared with pediatric and adult-onset IBD patients [7]. However, EOUC has also been reported in other studies as a predictive factor for increased morbidity and mortality [8, 9].

According to recent Japanese nationwide survey data, attention has been focused on the increasing number of EO cases. Komoto et al. [10] reported that EO patients show increased disease activity, with an increased proportion requiring UC-related hospitalization and UC-related surgery. Over the last decade, several new therapies for UC (immunosuppressors, biologics) have improved patient outcomes, and these treatments are included in international guidelines [11, 12]. Therefore many NEO patients avoid colectomy. However, as patients grow older, the need for colectomy increases due to colitis-associated colorectal cancer/dysplasia.

Thus, colectomy for UC in elderly patients is increasing [13], but there are few reports about elderly UC patients who underwent colectomy. There is no study comparing EO and NEO. The aim of this study was to analyze the differences in patient characteristics, preoperative medical treatment, surgical indications, and short-term outcomes, especially postoperative mortality, between EO and NEO patients aged 65 years and older who underwent UC-related surgery.

## Methods

### Inclusion and exclusion criteria

We identified 1973 patients with UC who underwent colectomy at Hyogo College of Medicine between January 1, 1984, and December 31, 2018. Only the data of patients who were 65 years old and older and underwent colectomy were retrospectively analyzed in this study (n = 221, 11.2%).

### Research methods

Patients were divided into two groups according to their age at disease onset: those younger than 60 years old (NEO) and those 60 years old and older (EO). The following data were retrospectively collected: age at surgery, sex, severity, preoperative medication, surgical indications, emergency surgery, postoperative complications (Clavien-Dindo classification grade ≧ III), and mortality. In this study, we compared these data between the two groups.

### Definitions

Acute severe colitis was defined according to Truelove and Witt’s criteria [14].

Surgery was determined as ‘elective’ if the decision to operate for UC was made prior to hospital admission, whereas the decision to perform ‘emergency’ colectomy was decided during or after admission on the basis of acute complications or for UC refractory to in-hospital intensive medical management.

Early postoperative complications were classified into 5 severity grades according to Dindo et al. [15]. In this study, postoperative complications occurred within 30 days after surgery and were classified as significant postoperative complications. Significant postoperative complication was defined as postoperative complication with Clavien-Dindo grade III or larger. Postoperative mortality was defined as death related to the surgical procedure during the first 30 postoperative days.

### Statistical analysis

All statistical analyses were carried out using JMP ver. 12 (SAS Institute, Inc., Cary, North Carolina, USA). Qualitative variables are expressed as frequencies and percentages. Quantitative variables are expressed as medians and ranges. The comparison of quantitative variables was performed by the Mann‐Whitney test. For qualitative variables, we used Chi‐square or Fisher’s exact tests. Multivariate logistic regression analysis was performed with the forward stepwise procedure. A value of p < 0.05 was considered statistically significant.

## Ethical considerations

This study was approved by the Institutional Review Board of the Hyogo College of Medicine (No. 202006-038).

## Results

### Patient backgrounds

The data of 221 patients who underwent colectomy 65 years old and older were retrospectively analyzed. Patient data in this study are presented in Additional file 1. Among them, 155 patients had EO, and 66 patients had NEO. Table 1 summarizes the characteristics of the elderly patients in this study. Sex and body mass index were not significantly different between the two groups, although the duration of disease was significantly shorter in the EO group than in the NEO group (245 months vs. 22 months, p < 0.01). There was no significant difference in the extent of colitis between the two groups, but the incidence of severe or fulminant type UC was significantly greater in the EO group than in the NEO group (p < 0.01).

**Table 1:** Patient characteristics (n = 300, NEO = 66/EO = 155)

	NEO (< 60) n = 66	EO (60 ≦) n = 155	p value
Sex (Male/Female)	35/31	101/54	0.1
Age at operation (years)	68 (65–83)	72(65–88)	< 0.01
Body mass index (kg/m^2^)	18.8 (12.7–34.5)	18.2 (13.5–28.8)	0.48
Duration of disease (months)	245 (76–580)	22 (0.5–258)	< 0.01
Extent of colitis (Pan-colitis/Left-side/Proctitis)	49/17/0	130/24/1	0.66
Severity (Mild/Moderate/Severe, Fulminant)	24/31/11	16/73/66	< 0.01

The data are shown as medians and ranges

### Preoperative medication

Preoperative medications are shown in Table 2. The preoperative total dose of steroids was significantly higher in the NEO group than in the EO group (8250 mg vs. 2634 mg, p < 0.01). In contrast, the preoperative daily dose of steroids was significantly higher in the EO group than in the NEO group (0 mg vs. 10 mg, p < 0.01). The rates of immunosuppressant, infliximab (IFX) and adalimumab use did not differ significantly between the groups. EO patients underwent cytapheresis much more frequently than NEO patients (24.2% vs. 38.7%, p = 0.04).

**Table 2:** Preoperative medication (n = 300, NEO = 66/EO = 155)

	NEO (< 60) n = 66	EO (60 ≦) n = 155	*p* value
Total corticosteroids dose (mg)	8250 (0–200,000)	2634 (0–100,000)	< 0.01
Daily corticosteroids dose (mg)	0 (0–60)	10 (0–80)	< 0.01
Immunomodulator use (%)	18 (27.3)	56 (36.1)	0.22
Infliximab use (%)	9 (13.6)	22 (14.2)	1
Adalimumab use (%)	2 (3.0)	9 (5.8)	0.51
Cytapheresis (%)	16 (24.2)	60 (38.7)	0.04

The data are shown as medians and ranges

### Surgical indications

The surgical parameters are shown in Table 3. The main surgical indication in NEO patients was colitis-associated cancer/dysplasia (32/66, 47%). In contrast, refractory medical treatment was the leading cause of surgery in EO patients (80/155, 52%). The distribution of surgical indications was different between the two groups (p < 0.01). Significantly more patients underwent emergency surgery in the EO group than in the NEO group (14% vs. 35%, p < 0.01).

**Table 3:** Surgical parameters (n = 300, NEO = 66/EO = 155)

	NEO (< 60) n = 66	EO (60 ≦) n = 155	p value
Surgical indication (%)			
Severe/Fluminant	9 (14)	54 (35)	< 0.01
Refractory medical treatment	25 (39)	80 (52)	
Colitis-associated colorectal cancer/Dysplasia	32 (47)	21 (13)	
Surgical setting (%)			
Elective	57 (86)	101 (65)	< 0.01
Emergency	9 (14)	54 (35)	

### Surgical procedure

In terms of surgical procedure, the EO group had 53 cases of total proctocolectomy, 62 cases of total colectomy, 36 cases of restorative proctocolectomy with IAA, and 4 cases of other procedure, while the NEO group had 20 cases of total proctocolectomy, 13 cases of total colectomy, 31 cases of restorative proctocolectomy with IAA, and 2 cases of other procedure. There was a significant difference between the two groups (p < 0.05).

### Postoperative complications

Table 4 shows postoperative complications. The postoperative morbidity was 17.4% (27/155) in the EO group and 13.6% (9/66) in the NEO group. There was no significant difference between the two groups. The most common postoperative complications were intrabdominal abscess in NEO patients and pneumonia in EO patients. In the EO group, there were 2 cases of residual rectum bleeding and 2 cases of duodenum bleeding. Other sources of bleeding were tumors and the inferior epigastric artery. Minor postoperative complications with Clavien-Dindo classification grade II ≤ were 46.9% (31/66) in NEO group and 45.1% (70/155) in EO group. The incidence of postoperative pneumonia was 7.9% (8/101) in the EO group and 1.7% (1/57) in the NEO group for elective surgery, and 16.6% (9/54) in the EO group and 0% (0/9) in the NEO group for emergency surgery.

**Table 4:** Postoperative complications (n = 18, Clavien-Dindo grade ≥ III)

	NEO (9/66, 13.6%)	EO (27/155, 17.4%)
Intra abdominal abscess	3	7
Pneumonia	0	8
Wound abscess	2	3
Bleeding	1	6
Bowel obstruction	3	1
Others	0	2

### Risk factors associated with postoperative complications

Table 5 shows the results of univariate analysis of risk factors associated with postoperative complications (Clavien-Dindo grade ≥ III). Emergency surgery (OR 8.00, 95% CI 3.70–17.2), Corticosteroids (OR 2.28, 95% CI 1.02–5.11) were risk factors. Table 6 shows the results of Multivariate analysis of risk factors associated with postoperative complications (Clavien-Dindo grade ≥ III). Emergency surgery (OR10.1, 95% CI 4.32–23.9), Corticosteroids (OR 2.43, 95% CI 0.98–6.04) were found to be an independent risk factors for postoperative complications (Clavien-Dindo grade ≥ III). Elderly onset. Elderly onset elderly UC patients tended to have more postoperative complications (OR 2.36, 95% CI 0.91–6.14), but there was no statistically significant difference (p = 0.07).

**Table 5:** Univariate analysis of risk factors associated with postoperative complications (Clavien-Dindo grade ≥ III)

	Incidence of Post operative complications (CD Grade III ≦)		OR	95% CI	p value
	Variable (+)	Variable (−)			
Elderly onset	27/155	11/66	1.05	0.48–2.27	0.89
Emergency surgery	26/63	12/158	8.00	3.70–17.2	< 0.01
Corticosteroids	29/136	9/85	2.28	1.02–5.11	0.03
Immunomodulator	10/74	28/147	1.15	0.46–2.85	0.30
Anti-TNF	7/37	31/184	0.66	0.30–1.45	0.76

**Table 6:** Multivariate analysis of risk factors associated with postoperative complications (Clavien-Dindo grade ≥ III)

	OR	95% CI	p value
Elderly onset	2.36	0.91–6.14	0.07
Emergency surgery	10.1	4.32–23.9	< 0.01
Corticosteroids	2.43	0.98–6.04	0.04
Immunomodulator	1.72	0.68–4.38	0.23
Anti-TNF	1.84	0.60–5.65	0.29

### Postoperative mortality

Postoperative mortality within 30 postoperative days was 5.8% (9/155) in the EO group and 1.5% (1/66) in the NEO group (Table 7). There were no significant differences between the two groups (p = 0.29). Additionally, we examined the mortality rate considering emergency or elective surgery. In elective cases, there were no significant differences between the two groups (p = 0.57). However, in the emergency setting, there were significant differences (p < 0.01). The prognosis of EO patients who underwent emergency surgery was extremely poor, and the mortality rate was 14.8% (8/54) (Table 8). The leading cause of postoperative death in EO patients who underwent emergency surgery was pneumonia (11.1%, 6/54). Sepsis caused by leakage from the stump following the Hartmann procedure and bleeding of the inferior epigastric artery were other causes of death.

**Table 7:** Causes of death (n = 300, NEO = 66/EO = 155)

	NEO (1/66, 1.5%)	EO (9/155, 5.8%)
Pneumonia	0	6
Sepsis (Intra abdominal abscess)	0	2
Bleeding	1	1

**Table 8:** Postoperative mortality (n = 300, NEO = 66/EO = 155)

	NEO (< 60) n = 66	EO (60 ≦) n = 155	p value
Total (%)	1 (1.5%)	9 (5.8%)	0.29
Elective surgery	1/57 (1.8%)	1/101 (1.0%)	0.57
Emergency surgery	0/9 (0%)	8/54 (14.8%)	< 0.01

## Discussion

It is well known that UC has a bimodal incidence distribution [1–6]. Especially in recent years, the rate of UC in elderly individuals has increased [16]. There are various reports on UC in elderly individuals. It was once reported that the disease course in elderly UC patients was relatively mild [17, 18]. However, the number of UC-related surgery cases in elderly individuals is increasing. Recently, it has been shown that elderly UC patients can be classified into two groups with different characteristics: those with EO and with NEO. Therefore, we compared these two groups in elderly UC patients who underwent surgery.

Regarding postoperative complications, there was no significant difference between the EO and NEO groups. Among the postoperative complications, infection accounted for 55% (5/9) in the NEO group and 66% (18/27) in the EO group. Preoperative steroid therapy has been reported to increase the risk postoperative infection [10], and preoperative medical treatment may have an impact on infection. Regarding perioperative mortality, there was no significant difference between the two groups among those who underwent elective surgery (p = 0.57). Among those who required emergency surgery, EO patients had a very high mortality rate of 14.5%, which was significantly different from that in NEO patients (p < 0.01). Increased mortality due to UC-related emergency surgery in elderly patients has previously been reported [19], but there were no reports comparing NEO and EO cases.

The most common cause of death was pneumonia. Accordingly, it is considered necessary to evaluate the preoperative respiratory system. Cautious treatment selection and moderate surgical intervention may be needed for patients with respiratory disease or a high risk of perioperative pneumonia. In Japan, the early and aggressive administration of immunosuppressants was started in the early 2000s. Tacrolimus (Tac) and biological therapies have been covered by insurance since 2009 and 2010, respectively. Especially in Japan, Tac and IFX are often used to induce remission of severe UC. In recent years, progress in the medical treatment of UC has been remarkable. With the advent of new drugs, medical treatment options are increasing. In fact, there are also new gut-targeted biologics, such as vedolizumab, which may have a more favorable safety profile and shift decision making toward medical therapy [20]**.** Although there are many cases in which young people may require second-line and third-line therapies, considering the prognosis of EO patients in this study, it is important to treat EO cases carefully so that emergency surgery can be avoided; it is important to recognize the appropriate timing for the transition to surgical treatment and not miss the optimal treatment window. In other words, in the case of EO, it is necessary to make a strict judgment regarding medical treatment not prolong the decision longer than necessary. In addition, physicians and surgeons should collaborate to treat EO patients to avoid errors in the timing of surgery. It may be important for EO patients to have a surgeon involved from the time of secondary treatment to discuss the appropriate timing of surgery. When surgery is deemed appropriate, performing surgery under elective conditions rather than under emergent conditions can substantially reduce mortality.

We previously reported that an Onodera’s prognostic nutritional index (10 × serum albumin (g/dL) + 0.005 × total lymphocyte count) [21] of 25 or less is a risk of postoperative mortality in UC patients and that the mortality rate was 13.6% (6/44) [22]. This index may be a useful indicator of surgery timing. In addition, It has also been reported that this index value of less than 35 is a risk factor for postoperative pouch-related complications [21]. This index may be useful to make decisions in selecting surgical techniques for high-risk emergency cases. For high-risk patients, it may be important to choose a surgical technique in which the initial surgery is a total colectomy only, with no reconstruction. This study has some limitations. First, this was a retrospective study. Second, our facility is an IBD-specialty hospital, and the patient population may be different from that in the real world. Third, old data were included in this study, and there were missing data on the underlying diseases of patients; thus, it was not possible to evaluate these.

## Conclusion

Among UC elderly patients who underwent UC-related surgery, the outcomes of emergency surgery in EO patients were very poor compared with those in NEO patients; the mortality rate was 14.8%. Emergency surgery and preoperative corticosteroids use were found to be an independent risk factors for postoperative complications (Clavien-Dindo grade ≥ III). The most common cause of death was pneumonia. In such cases, it is important for the physician and surgeon to begin communication at an early stage so that the optimal timeframe for surgery is not missed.

## Supplementary Information

**Additional file 1.** Patient data in this study.

## Data Availability

The data used in this study are shown in the additional materials.

## Abbreviations

UC: Ulcerative colitis
EO: Elderly-onset UC
NEO: Nonelderly-onset UC
Tac: Tacrolimus
